# Determination of the Soluble Solids Content in Korla Fragrant Pears Based on Visible and Near-Infrared Spectroscopy Combined With Model Analysis and Variable Selection

**DOI:** 10.3389/fpls.2022.938162

**Published:** 2022-07-06

**Authors:** Xuhai Yang, Lichun Zhu, Xiao Huang, Qian Zhang, Sheng Li, Qiling Chen, Zhendong Wang, Jingbin Li

**Affiliations:** ^1^Xinjiang Production and Construction Corps, Key Laboratory of Modern Agricultural Machinery, College of Mechanical and Electrical Engineering, Shihezi University, Shihezi, China; ^2^Xinjiang Production & Construction Crops, Key Laboratory of Korla Fragrant Pear Germplasm Innovation and Quality Improvement and Efficiency Increment, Shihezi, China

**Keywords:** portable spectral measurement, internal attribute evaluation, Korla fragrant pear, variable selection, quantitative analysis model

## Abstract

The non-destructive detection of soluble solids content (SSC) in fruit by near-infrared (NIR) spectroscopy has a good application prospect. At present, the application of portable devices is more common. The construction of an accurate and stable prediction model is the key for the successful application of the device. In this study, the visible and near-infrared (Vis/NIR) spectra of Korla fragrant pears were collected by a commercial portable measurement device. Different pretreatment methods were used to preprocess the raw spectra, and the partial least squares (PLS) model was constructed to predict the SSC of pears for the determination of the appropriate pretreatment method. Subsequently, PLS and least squares support vector machine (LS-SVM) models were constructed based on the preprocessed full spectra. A new combination (BOSS-SPA) of bootstrapping soft shrinkage (BOSS) and successive projections algorithm (SPA) was used for variable selection. For comparison, single BOSS and SPA were also used for variable selection. Finally, three types of models, namely, PLS, LS-SVM, and multiple linear regression (MLR), were constructed based on different input variables. Comparing the prediction performance of all models, it showed that the BOSS-SPA-PLS model based on 17 variables obtained the best SSC assessment ability with *r*_p_ of 0.94 and *RMSEP* of 0.27 °Brix. The overall result indicated that portable measurement with Vis/NIR spectroscopy can be used for the detection of SSC in Korla fragrant pears.

## Introduction

Fruit is one of the most important foods in people’s daily life. Fruit industry is a pillar industry in many countries and regions. The post-harvest quality detection and grading can realize the graded sales of fruit in the market, which not only greatly increases the profits but also improves the market competitiveness (Londhe et al., 2013). At present, many grading equipment manufacturers have successfully developed commercial systems for the quality detection of fruit. Fruit quality includes external and internal qualities. Compared with external quality, such as size, color, and shape, consumers prefer fruits with good internal quality, because it is directly related to taste. Soluble solids content (SSC) is an important internal quality attribute that affects consumers’ acceptance and price of fresh fruit. It is also an important index for determining fruit maturity and harvest time, as well as for evaluating and grading fruit post-harvest quality (Antonucci et al., 2011; Rajkumar et al., 2012). Non-destructive testing of SSC in fruit by refractometer is a standard detection way, which is destructive, cumbersome, and time-consuming (Li and Chen, 2017). This way is only suitable for detecting a small amount of fruit in specific circumstances, such as sampling inspection. However, for quality assessment of a large number of fruit, the rapid and non-destructive measurement techniques are more attractive.

In the past three decades, many technologies have been applied to detecting the SSC in fruits (Li et al., 2016; Walsh et al., 2020). Among them, the visible and near-infrared (Vis/NIR) spectroscopy is the most widely used technology. The detected fruits include apple (Ma et al., 2021), orange (Jamshidi et al., 2012), pear (Li et al., 2013), jujube (Wang et al., 2011), watermelon (Ali et al., 2017), melon (Zhang et al., 2019), banana (Zude, 2003), etc. For the detection of SSC in fruits by Vis/NIR spectroscopy, the way of measurement can be divided into static, online, and portable detection. In the early stage, the static detection was the most commonly used way using expensive testing instrument, which was mainly aimed at verifying the feasibility of Vis/NIR spectroscopy to detect the SSC of fruit and constructing appropriate prediction models. On this basis, many studies have proved that Vis/NIR spectroscopy was an effective technology for the SSC analysis of fruits (Walsh et al., 2020). Therefore, this study mainly focuses on the online SSC detection for developing a suitable prediction model for processing large quantities of fruit (Xia et al., 2020; Zhang et al., 2021). Different from the static and online detections, the portable detection is a rapid detection technique for assessing the internal quality of fruits based on portable measuring instruments (Neto et al., 2017). This way of detection has the unique advantages of convenient carrying and flexible use. This way is more suitable for the SSC inspection of fruit at anytime and anywhere in the process of storage and transportation and is also suitable for the detection of fruit maturity on trees and so on. In terms of these three ways of detection, no matter which way needs to build a special prediction model for different varieties of fruits to accurately predict the SSC, because of still many problems in the model transfer between different ways of detection and between prediction models of different varieties of fruits (Mishra et al., 2021).

The prediction model of SSC based on Vis/NIR spectroscopy contains linear [such as partial least squares (PLS) and multiple linear regression (MLR)] and non-linear [least squares-support vector machine (LS-SVM) and artificial neural network (ANN)] models, which can achieve the successful prediction of SSC in fruits (Walsh et al., 2020). Due to the different application objects and conditions, it is difficult to directly determine which model is better without actual verification. Generally, compared with non-linear models, the linear models are easier to explain and are simpler. However, the non-linear models may be more robust because they can deal with the linear and non-linear relationship between spectral data and prediction attributes at the same time (Li et al., 2013). However, this cannot be the judgment basis for using linear and non-linear models in actual SSC prediction. To find the best prediction model, it is necessary to build different models for analysis.

In the process of development, model optimization is the key to build a more efficient prediction model. Variable selection is a common model optimization strategy (Zou et al., 2010; Yun et al., 2019). By using appropriate variable selection methods, those uninformative variables and redundant variables are eliminated, and a small number of variables related to SSC prediction can be extracted, so as to achieve the purpose of model optimization. The variable selection can make the model simpler and improve the interpretation, modeling, and prediction rate of the model. For model optimization, many variable selection methods [such as successive projections algorithm (Araújo et al., 2001), competitive adaptive reweighted sampling (Li et al., 2009), and Monte Carlo uninformative variable elimination (Cai et al., 2008)] have been successfully applied. Compared with the variable selection using single method, some studies in fruit quality detection indicated that two complementary wavelength selection strategies may achieve a superimposed effect when combined together (Li et al., 2014). Therefore, in this study, a new combination (BOSS-SPA) of bootstrapping soft shrinkage (BOSS) and successive projections algorithm (SPA) will be applied to select the effective variables from full spectral data.

Pear is among the economically most important fruit in the world. The main objective of this study was to determine the best model for SSC prediction of Korla fragrant pears based on portable spectral detection technology. The specific purposes were given as follows: (1) To collect Vis/NIR spectral data of all pear samples using a commercially available portable spectroscopic device; (2) To establish the linear PLS and non-linear LS-SVM calibration models based on full-spectrum data and compare the performance of models; (3) To extract the effective variables that were most informative for SSC detection of Korla fragrant pears by using BOSS-SPA combination variable selection method; and (4) To determine the optimal predictive model, combined with prediction accuracy and stability, by comparing the performance of models established based on full spectra and effective variables.

## Materials and Methods

### Fruit Samples

Korla fragrant pear, a unique variety in Xinjiang, China, was used in this study. A total of 120 intact pears were purchased from a grocery store. All samples were returned to the laboratory and stored at room temperature (20°C, relative humidity 60%) for 24 h, to avoid the influence of sample temperature on the accuracy of the prediction model (Xia et al., 2020). In this study, all samples were divided into calibration set and prediction set on the basis of Kennard-Stone (KS) sampling method (Galvão et al., 2005). The calibration set contained 80 samples, which were mainly used for the construction of models. The prediction set contained 40 samples, which were mainly used to evaluate the performance of models. In the whole process of data analysis, the samples of calibration set and prediction set remain unchanged.

### Portable Measurement Device for Spectral Data Acquisition

A commercial portable spectrometer (K-BA100R; Kubota Co., Osaka, Japan) was used to collect Vis/NIR spectral data of samples. This portable measurement device mainly contains halogen lamp light source, ring detection probe, optical fiber, display screen, processor, etc. The detection probe consists of two groups of ring optical fibers. One is the transmitting optical fiber, which is mainly used to transmit Vis/NIR light to the sample; and the other is the receiving optical fiber, which is mainly used to receive the diffuse reflectance light with fruit component information. Spectral data were acquired based on interactive mode. During spectral data collection, each sample was placed on the detection probe with its stem-calyx axis being horizontal. The collected spectral range was 500–1,010 nm with an interval of 2 nm. The integration time of spectrum acquisition was set to 300 ms for each sample. The final spectrum (*Rc*) was calculated automatically by using the raw sample spectrum (*R*), the dark reference spectrum (D), and the white reference spectrum (W), according to *Rc* = [(*R* – *D*)/(*W* – *D*)]. The dark spectrum and the white spectrum were obtained by turning off and turning on light sources (no sample information), respectively. Due to the noise at both ends of the original spectrum, only spectral data in the range of 550–1,000 nm were used.

### Real Soluble Solids Content Measurement

After the spectral data of all samples were collected, the actual SSC was measured immediately. A commercial refractometer (Model: PR-101α, Atago Co., Ltd., Tokyo, Japan) with a refractive index accuracy of ±0.1 and the range of 0–45% with temperature correction was used for destructive measurement. For each sample, the whole fruit was juiced, and the SSC value of the juice was measured three times. The mean values of three measurements were recorded as the actual SSC value of the tested sample.

### Wavelength Selection Methods

The original spectrum contains over 200 wavelengths (variables), not all of which are related to the prediction of SSC in pears, and moreover, too many wavelengths are not conducive to the construction of robust model. This study used the BOSS-SPA combination to extract the effective wavelengths from full spectral data. In terms of the BOSS-SPA combination, BOSS was first used to extract a set of effective wavelengths, and SPA was then used to optimize the extracted wavelengths. BOSS method, originally proposed by Deng et al. (2016), takes advantage of bootstrap sampling (BSS) and weighted bootstrap sampling (WBS) to generate random variable subsets for the construction of partial least squares regression (PLSR) sub-models. The regression coefficients of sub-models were analyzed, and the weights of variables were determined according to the absolute values of the regression coefficients. The informative variables with higher weights have a higher selection probability. Model population analysis (MPA), proposed by Deng et al. (2015), was used to analyze the sub-models to update the weight of variables. Variables were optimized according to the principle of soft shrinkage; in other words, less important variables were not eliminated directly, but assigned smaller weights. The algorithm iterates until the number of variables reaches 1. The subset with the lowest root mean square error of cross validation (RMSECV) was finally selected as the optimal variable set. SPA proposed by Araújo et al. (2001) is a forward wavelength selection algorithm, which aims to minimize the collinearity problem in variables. SPA uses a simple projection operation in a vector space to obtain a subset of wavelengths with minimal collinearity. The final selected variable set corresponds to the smallest root mean square error of prediction (RMSEP) in MLR analysis.

### Modeling Algorithms

The PLS has become the most commonly used multivariate linear analysis method in spectral modeling and analysis. In the process of modeling, PLS can consider the target value matrix Y (SSC value in this study) and spectral matrix X at the same time and establish the basic relationship between X and Y. For the development of a PLS model, the spectral matrix X and the concentration matrix Y were first decomposed to obtain the corresponding score matrices T and U:


(1) X=TP+E,Y=UQ+F

where P and Q are the loading matrices of X matrix and Y matrix, and E and F are the errors that come from the process of PLS. Then, MLR based on score matrix T and U was performed as follows:


(2) U=BT+E

where B is the regression coefficient matrix of PLS. In linear regression, it is necessary to consider how many columns of data in the T matrix, i.e., the best factor or later variables (LVs), were used for modeling. In this study, the leave-one-out cross validation was used to determine the number of optimal LV.

The LS-SVM is an advanced statistical learning method, which can deal with linear and non-linear multivariate analysis and solve these problems in a relatively fast way. The LS-SVM regression model can be expressed as follows:


(3) $$y\,\left(x\right)=\sum_{k=1}^{N}\alpha_{k}\,K\,\left(x,x_{k}\right)+b$$

where *K*(*x*, *x*_*k*_), *x*_*k*_, *α_*k*_*, and *b* are the kernel function, input vector, support value, and bias, respectively. The radial basis function (RBF) was used as kernel function *K*(*x*, *x*_*k*_) in this study and defined as follows:


(4) K(x,x)k=exp(-||x-kx||/2(2σ)2)

where ——*x*_*k*_-*x*—— represents the distance between the input vector and the threshold vector, and σ is a width vector.

The MLR is also a common calibration method for spectral quantitative analysis, which is easy to calculate and explain compared with PLS. The general form of the model is:


(5) y=β⁢x+b

where y represents an unknown concentration value (here, it was the SSC value), β represents a set of regression coefficients, x represents the spectral vector of a sample, and b is a constant. MLR is suitable for a simple system with good linear relationship. However, MLR also has the limitation. This method requires more samples than variables for modeling. In practical applications, the raw spectral variables obtained by spectrometers are often numerous. Therefore, before constructing MLR models, it is usually necessary to use the wavelength selection method to optimize the variables to meet the prerequisite condition of MLR modeling.

### Model Evaluation

Four parameters, including calibration correlation coefficient (*r*_c_) and root mean square error of calibration (*RMSEC*), and prediction correlation coefficient (*r*_p_) and root mean square error of prediction (*RMSEP*), were used to assess the performance of models. The first two parameters were used to evaluate the prediction performance of models on the samples in the calibration set, and the last two parameters were used to evaluate the prediction performance of models on the samples in the prediction set. A good model usually has high *r*_c_ and *r*_p_, low *RMSEC* and *RMSEP*, and a small difference between *RMSEC* and *RMSEP*. All parameters were calculated as follows:


(6) $$r=\sqrt{1-\frac{\sum_{i=1}^{n}{\left(y_{i,a\,c\,t\,u\,a\,l}-y_{i,p\,r\,e\,d\,i\,c\,t\,e\,d}\right)}^{2}}{\sum_{i=1}^{n}{\left(y_{i,a\,c\,t\,u\,a\,l}-{\bar{y}}_{i,a\,c\,t\,u\,a\,l}\right)}^{2}}}$$

(7) $$R\,M\,S\,E\,C=\sqrt{\frac{1}{n_{c}}\,\sum_{i=1}^{n_{c}}{\left(y_{i,p\,r\,e\,d\,i\,c\,t\,e\,d}-y_{i,a\,c\,t\,u\,a\,l}\right)}^{2}}$$

(8) $$R\,M\,S\,E\,P=\sqrt{\frac{1}{n_{p}}\,\sum_{i=1}^{n_{p}}{\left(y_{i,p\,r\,e\,d\,i\,c\,t\,e\,d}-y_{i,a\,c\,t\,u\,a\,l}\right)}^{2}}$$

where _*y_i,predicted_*_ and *y*_*i,actual*_ are the predictive SSC value and the real SSC value of the *i*th sample in the calibration set or prediction set, respectively. ${\bar{y}}_{i,a\,c\,t\,u\,a\,l}$ is the average SSC value of samples in the calibration or prediction set. *n*_c_, *n*_p_, and *n* correspond to the number of calibration samples, prediction samples, and all samples, respectively.

## Results and Discussion

### Analysis of Soluble Solids Content Values of All Samples

Table 1 shows the statistical results of SSC values (°Brix) of all samples. It can be seen that the maximum, minimum, mean, and standard deviation (S.D.) of SSC values for 90 samples of calibration set were 14.5, 11.0, 12.6, and 0.6 °Brix, respectively, and for 30 samples of prediction set, these four values were 14.3, 11.2, 12.5, and 0.8 °Brix, respectively. The SSC range of the calibration set covers that of the prediction set, which is helpful to build a more robust prediction model.

**TABLE 1:** The statistics of SSC (°Brix) of all samples.

Data set	No. of samples	Min.	Max.	Mean	S.D.
Total	120	11.0	14.5	12.6	0.8
Calibration set	90	11.0	14.5	12.6	0.8
Prediction set	30	11.2	14.3	12.5	0.6

### Spectral Pretreatment and Spectral Features

The difference of sample size leads to large scattering in the original spectra, and the original spectra can also contain random noise, which negatively affects the prediction performance of the model. Therefore, the original spectrum was preprocessed before model construction. The pretreatments, including Savitzky-Golay smoothing (SG), first derivative and second derivative, combination of SG and standard normal variables (SG-SNV), combination of SG and multivariate scattering correction (SG-MSC), and combination of derivative and SG-MSC, were used for spectral pretreatment. Table 2 shows the prediction results of SSC by PLS models combined with preprocessing and raw spectra. It can be seen that the prediction accuracy of all PLS models based on the preprocessed spectra was better than that of the PLS model based on the original spectra, indicating that the spectrum preprocessing can improve the prediction performance of the model. PLS models combined with SG-MSC and the first derivative-SG-MSC preprocessing achieved the best prediction results. Compared with the second derivative-SG-MSC preprocessing, the performance of the first derivative-SG-MSC is better, probably because the second derivative processing amplifies the noise in the original spectrum. For samples of the prediction set, the optimal *r*_p_ and *RMSEP* were 0.92 and 0.25, respectively. Considering that SG-MSC pretreatment is simpler than the first derivative-SG-MSC, the pretreatment spectra by SG-MSC were used for the subsequent analysis.

**TABLE 2:** Prediction results of SSC by PLS models combined with different preprocessing methods.

Preprocessing methods	LVs	Calibration set		Prediction set	
		*r*_c_	*RMSEC*	*r*_p_	*RMSEP*
None	7	0.97	0.19	0.86	0.32
SG	10	0.97	0.20	0.91	0.27
SG-MSC	11	0.97	0.20	0.92	0.25
SG-SNV	10	0.96	0.22	0.89	0.29
First derivative-SG-MSC	11	0.96	0.21	0.92	0.25
Second derivative-SG-MSC	12	0.93	0.25	0.90	0.27

**FIGURE 1:**
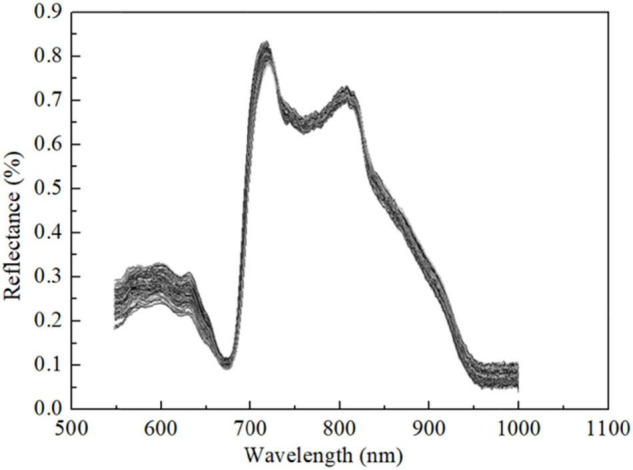
Preprocessed spectral curves by SG-MSC.

The preprocessed spectral curves of samples by SG-MSC are shown in Figure 1. It can be seen that all samples have a similar spectral trend in the Vis-NIR spectral region of 550–1,000 nm, and there are no abnormal samples. The spectral curve shows some obvious absorption and reflection peaks, which may be related to the internal chemical components of Korla fragrant pears. The first obvious absorption peak is about 680 nm, which is a typical chlorophyll absorption band. The central band of the second absorption peak is about 750 nm, which is a relatively wide absorption band associated with the fourth overtone of band C–H. The small absorption band around at 950 nm might be associated with the second overtone of band O–H. These results were similar to those of Li et al. (2018). In addition to the typical absorption characteristics, the spectral intensities of different samples were different, indicating that there were differences between chemical components, which was conducive to construct the SSC quantitative analysis model.

### Full Spectra Models for Soluble Solids Content Prediction

In this study, two kinds of full-spectrum models, namely, linear PLS and non-linear LS-SVM were constructed to predict the SSC of pears. Prediction results are shown in Table 3. It can be seen that the prediction accuracy of PLS model was obviously better than that of LS-SVM model. For samples in the prediction set, the *r*_p_ and *RMSEP* of the latter were 0.92 and 0.25, respectively. The relatively high prediction accuracy indicated that the PLS model seems to be more suitable for the non-destructive evaluation of SSC of Korla fragrant pears, which may be due to the main linear relationship between the original spectral data and SSC of fragrant pears. For the PLS model, the optimal number of potential variables (LVs) was 11. Nevertheless, full variable modeling negatively influences the fast construction of the model and also reduces the prediction efficiency of the model.

**TABLE 3:** Prediction results of SSC by PLS and LS-SVM with full spectral data, respectively.

Modeling methods	LVs/(γ/σ^2^)	Calibration set		Prediction set	
		*r*_c_	*RMSEC*	*r*_p_	*RMSEP*
PLS	11	0.97	0.20	0.92	0.25
LS-SVM	γ = 2.1 × 10^5^; *σ^2^* = 2.5 × 10^4^	0.95	0.24	0.88	0.32

### Wavelength Selection by Bootstrapping Soft Shrinkage and Successive Projections Algorithm

**FIGURE 2:**
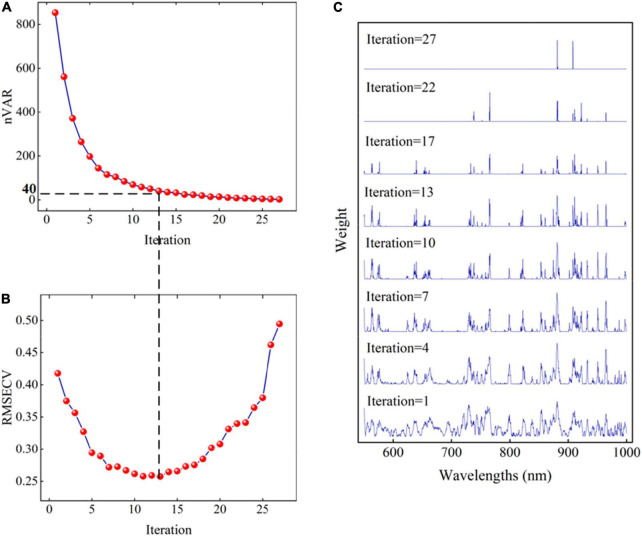
The change of nVAR **(A)**, RMSECV **(B)**, and weights for variables **(C)** in each iteration of the BOSS algorithm.

The BOSS-SPA combination algorithm was used to select the most important wavelengths from all 450 spectral variables to build a more efficient SSC prediction model. The process of wavelength selection by the BOSS algorithm is shown in Figure 2. The evolution of wavelength number (nVAR), RMSECV, and weights in sub-models in each iteration of BOSS are shown in Figures 2A–C, respectively. As shown in Figure 2A, the number of variables shows a downward trend from fast to slow with the increase in the number of iterations. However, it is impossible to know how many variables are finally selected. It can be seen from Figure 2B that the number of the selected variables is directly related to the RMSECV value of the models. Observing the RMSECV curve, combined with Figure 2A, indicates that the prediction performance of the model gradually improves with the decrease in the number of selected variables. When the number of selected variables reaches 40 (the corresponding number of iterations is 13), the lowest RMSECV value was obtained. Afterward, the RMSECV value of the model begins to increase rapidly with the increase in the number of selected variables, indicating that the performance of the model gradually deteriorates. Therefore, the 40 variables corresponding to the lowest RMSECV value were considered as the most important wavelengths, which were selected by the BOSS algorithm. Figure 2C shows the change of each wavelength weight in different iterations. It can be seen that the extracted 40 wavelengths were distributed in the Vis/NIR spectrum region. This showed that the tissue color of Korla fragrant pears, especially the skin color, may have a certain correlation with SSC.

**FIGURE 3:**
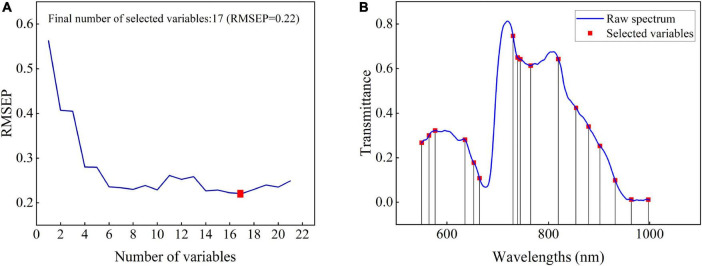
The change of RMSEP with the selected variables by SPA **(A)** and distribution of 17 variables **(B)**.

Although the selected 40 wavelengths account for only 8.9% of the full spectrum variable information, it can be seen from the weight figure that there is obvious collinearity between wavelengths, that is, there are more redundant variables in the selected variables. Thus, based on the selected 40 wavelengths, SPA was further used to optimize variables. During variable selection by SPA, the variation of RMSEP of the MLR model with the used variable number is shown in Figure 3A. The red solid block in the figure indicates the optimal number of the selected variables by SPA. It indicates that only 17 wavelengths are selected from 40 spectral variables. The number of variables is further reduced. The selected 17 wavelengths include 550, 565, 577, 636, 653, 664, 730, 739, 744, 765, 819, 854, 880, 902, 932, 966, and 997 nm, as shown in Figure 3B. In Figure 3B, the vertical line represents the positions of the corresponding 17 wavelengths. For these selected wavelengths, the first nine wavelengths are located in the visible spectrum region, which are mainly related to the color characteristics of the pear surface. The other eight wavelengths are located in the NIR spectral region of 750–1,000 nm. The absorbance of this region was related to the second and third overtones of oxygen–hydrogen (O–H) stretches and the third and fourth overtones of carbon–hydrogen (C—H) stretches of the organic molecules such as SSC (Liu et al., 2010; Jie et al., 2013; Li and Chen, 2017).

### Effective Variable Models for Soluble Solids Content Prediction

Three kinds of models, namely, PLS, LS-SVM, and MLR, were established based on selected variables by BOSS-SPA for SSC prediction of Korla fragrant pears. For comparison, three types of models were also constructed based on those variables selected by only using BOSS or SPA method. Note that because the SPA variable selection process based on full spectrum is similar to SPA in the BOSS-SPA combination variable selection method, it is further introduced in this study; 24 variables were selected by only using SPA. Prediction results of all models are shown in Table 4. It can be seen that all models can effectively predict the SSC of pears, and the *r*_p_ and *RMSEP* ranges of models were 0.89–0.93 and 0.23–0.32 °Brix, respectively. Compared with full-spectrum PLS and LS-SVM models in Table 3, the models based on effective variables obtained similar or even better prediction performance. The results showed that the appropriate variable selection method can optimize the model. Comparing the three types of models (PLS, LS-SVM, and MLR) in Table 4, it can be found that the prediction accuracy of the two types of linear models for SSC was slightly better than that of the LS-SVM model based on the same inputs, indicating that the linear model was a better choice when a portable instrument was used to measure SSC of Korla fragrant pears. In terms of PLS and MLR models, the prediction accuracy of the two models was similar. For each type of model in PLS, MLR, and LS-SVM shown in Table 4, the models (i.e., BOSS-PLS, BOSS-LS-SVM, and BOSS-MLR) developed based on the variables selected by BOSS were the best, followed by the models (i.e., BOSS-SPA-PLS, BOSS-SPA-LS-SVM, and BOSS-SPA-MLR) developed based on the variables selected by BOSS-SPA. The prediction ability of the models (i.e., SPA-PLS, SPA-LS-SVM, and SPA-MLR) developed based on the variables extracted by the SPA algorithm was the worst, which may be because SPA can effectively reduce the collinearity between variables, but it is weak in the elimination of uninformative variables. Therefore, there may be uninformative variables in those variables selected by SPA. In contrast, the BOSS algorithm can effectively eliminate those uninformative variables. The BOSS-SPA combination variable selection method takes into account the advantages of both BOSS and SPA. Based on BOSS-SPA, only 17 variables were selected, and the models based on these selected variables achieved high prediction accuracy for the SSC prediction of Korla fragrant pears. For samples in the calibration set, the *r*_c_ and *RMSEC* of BOSS-SPA-PLS, BOSS-SPA-LS-SVM, and BOSS-SPA-MLR models were 0.94 and 0.27 °Brix, 0.96 and 0.21 °Brix, and 0.94 and 0.25 °Brix, respectively. For samples in the prediction set, the *r*_p_ and *RMSEP* were 0.92 and 0.25 °Brix, 0.91 and 0.28 °Brix, and 0.92 and 0.25 °Brix, respectively, for the three models.

**TABLE 4:** Prediction results of SSC by PLS, LS-SVM, and MLR models with different effective wavelengths.

Models	Variable selection methods	LVs/(γ/σ^2^)	No. of variables	Calibration set		Prediction set	
				*r*_c_	*RMSEC*	*r*_p_	*RMSEP*
PLS	BOSS-SPA	8	17	0.94	0.27	0.92	0.25
	BOSS	9	40	0.96	0.23	0.93	0.23
	SPA	14	24	0.92	0.28	0.90	0.27
LS-SVM	BOSS-SPA	γ = 2.6 × 10^4^; *σ^2^* = 4.6 × 10^3^	17	0.96	0.21	0.91	0.28
	BOSS	γ = 5.3 × 10^4^; *σ^2^* = 5.1 × 10^3^	40	0.98	0.17	0.92	0.26
	SPA	γ = 7.3 × 10^5^; *σ^2^* = 8.6 × 10^4^	24	0.90	0.35	0.89	0.29
MLR	BOSS-SPA	—	17	0.94	0.25	0.92	0.25
	BOSS	—	40	0.94	0.25	0.92	0.23
	SPA	—	24	0.92	0.24	0.89	0.32

### Determination of the Optimal Model

**FIGURE 4:**
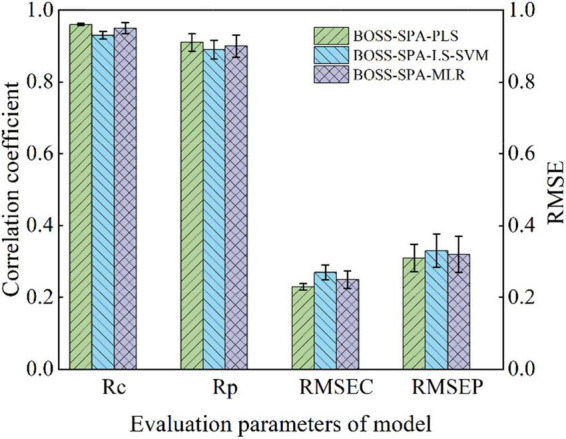
Results of 20 predictions for BOSS-SPA-PLS, BOSS-SPA-LS-SVM, and BOSS-SPA-MLR models.

The analysis in the Section “Effective Variable Models for Soluble Solids Content Prediction” shows that BOSS-SPA-PLS, BOSS-SPA-LS-SVM, and BOSS-SPA-MLR models have high prediction accuracy and few input variables, which can be used for the SSC evaluation of Korla fragrant pears. To further compare the prediction performance of the three models, the stability of the models was analyzed. Specifically, all 120 samples were randomly divided into calibration set and prediction set according to the ratio of 3:1, and then, BOSS-SPA-PLS, BOSS-SPA-LS-SVM, and BOSS-SPA-MLR models were constructed, respectively, based on the new sample set to predict SSC. The sample set was divided 20 times, and accordingly, each type of model was also constructed 20 times. Figure 4 shows the prediction results of 20 model calculations for the three types of models. For each type of model, the bar graph represents the average of the 20 predictions, and error bars from the 20 calculations were also shown on the bar graph. It can be observed from the figure that the BOSS-SPA-PLS model was optimal with the highest *r*_c_/*r*_p_ average and the lowest *RMSEC*/*RMSEP*. Moreover, the correlation coefficient and root mean square error (RMSE) of the BOSS-SPA-PLS model have the smallest change of error bar, indicating that this model has the highest stability for the SSC prediction. Therefore, the BOSS-SPA-PLS model was finally confirmed as the optimal model for predicting the SSC of Korla fragrant pears based on portable Vis/NIR spectroscopy.

**FIGURE 5:**
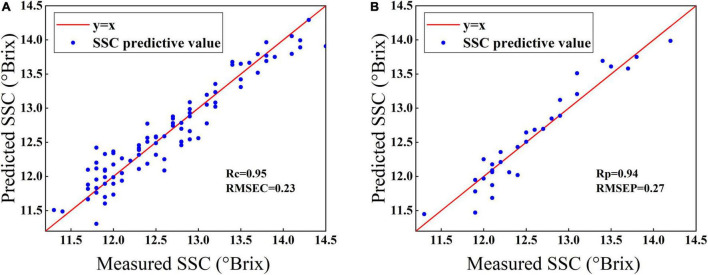
Measured vs. predicted values for SSC prediction of Korla fragrant pears by BOSS-SPA-PLS models. **(A)** Samples in the calibration set and **(B)** samples in the prediction set.

Figure 5 shows the scatterplots of the predicted vs. measured SSC values for calibration samples (Figure 5A) and prediction samples (Figure 5B) by the BOSS-SPA-PLS model. The red solid line is the regression line corresponding to the ideal prediction result. It can be observed that the samples were closely distributed around the regression line. The prediction accuracy of the model was 0.95 for *r*_c_ and 0.23 for *RMSEC* for samples in the calibration set and 0.94 for *r*_p_ and 0.27 for *RMSEP* for samples in the prediction set. Both *RMSEC* and *RMSEP* were low, and the difference between them was small, indicating that the BOSS-SPA-PLS model has a good prediction accuracy and stability, and it can be used to effectively predict the SSC of Korla fragrant pears.

Some similar studies have been carried out using portable Vis-NIR or NIR instruments. Sun et al. (2009) developed a portable NIR system to detect SSC of Nanfeng mandarin. The best results were obtained by the support vector machine model. The correlation coefficient (*R*) and *RMSEP* were 0.93 and 0.65 °Brix, respectively. Wang et al. (2017) achieved a prediction accuracy of 0.46 °Brix (*RMSEP*) for SSC analysis of the European pear based on the MLR model with 9 wavelengths. Fan et al. (2017) used Vis-NIR portable instrument to measure the SSC of apple and constructed a PLS model based on 50 wavelengths to obtain the best prediction performance, with *r*_p_ and *RMSEP* being 0.96 and 0.40 °Brix, respectively. Compared with these studies, satisfactory results were obtained in this study.

## Conclusion

In this study, the portable Vis/NIR device was successfully used to evaluate the SSC of Korla fragrant pears. It was found that SGS-SNV spectral preprocessing can obviously improve the prediction performance of models developed using the raw spectra. The PLS and LS-SVM models with full spectra were constructed. For samples in the prediction set, the *r*_p_ and *RMSEP* of the two models were 0.92, 0.25 °Brix and 0.88, 0.32 °Brix, respectively. Furthermore, to reduce the number of variables involved in modeling, the BOSS-SPA combination method selected 17 optimal variables, which were used to develop BOSS-SPA-PLS, BOSS-SPA-LS-SVM, and BOSS-SPA-MLR models. Moreover, PLS, LS-SVM and MLR models were also constructed based on the variables selected by the only BOSS and SPA. The results showed that the prediction accuracy of models with effective variables was similar or better than that of the full-spectrum models, and the ranges of *r*_p_ and *RMSEP* of models were 0.89–0.93 and 0.23–0.32 °Brix, respectively, for SSC prediction. For each model of PLS, LS-SVM, and MLR established based on the selected variables, BOSS-SPA-PLS, BOSS-SPA-LS-SVM, and BOSS-SPA-MLR were optimal by considering the complexity and accuracy of the models. The *RMSEP* values of the three models for SSC prediction of Korla fragrant pears were 0.25, 0.28, and 0.25 °Brix, respectively. The stability of the three models was further compared based on 20 modeling calculations, which showed that BOSS-SPA-PLS was superior to BOSS-SPA-LS-SVM and BOSS-SPA-MLR models. Finally, the BOSS-SPA-PLS was determined to be the best model, and the BOSS-SPA combination method was proved to be an effective variable selection method. The model developed in this study, combined with portable measurement technology, has the potential to be used for the non-destructive evaluation of SSC in Korla fragrant pears.

## Data Availability Statement

The original contributions presented in this study are included in the article/supplementary material, further inquiries can be directed to the corresponding authors.

## Author Contributions

XY: methodology, original manuscript writing, and funding. LZ and SL: modeling. XH: spectrum processing. QZ: funding, supervision, revision, and editing. QC: spectral pretreatment. ZW: editing. JL: revision, editing, and supervision. All authors contributed to the article and approved the submitted version.

## Conflict of Interest

The authors declare that the research was conducted in the absence of any commercial or financial relationships that could be construed as a potential conflict of interest.

## Publisher’s Note

All claims expressed in this article are solely those of the authors and do not necessarily represent those of their affiliated organizations, or those of the publisher, the editors and the reviewers. Any product that may be evaluated in this article, or claim that may be made by its manufacturer, is not guaranteed or endorsed by the publisher.
